# Primary thymic adenocarcinoma with an aggressive clinical course: An autopsy case showing signet ring cell‐like features

**DOI:** 10.1111/1759-7714.13700

**Published:** 2020-10-12

**Authors:** Ayako Shiono, Takashi Fujino, Kyoichi Kaira, Tomomi Kato, Masanori Yasuda, Kunihiko Kobayashi, Hiroshi Kagamu

**Affiliations:** ^1^ Department of Respiratory Medicine, Comprehensive Cancer Center, International Medical Center Saitama Medical University Saitama Japan; ^2^ Department of Pathology, Comprehensive Cancer Center, International Medical Center Saitama Medical University Saitama Japan

**Keywords:** Adenocarcinoma, lymphangitic carcinomatosis, pulmonary tumor thrombotic microangiopathy, signet ring cell‐like, thymic cancer

## Abstract

Thymic adenocarcinoma is an extremely rare neoplasm, and little is known about its pathogenesis and clinical characteristics. A 52‐year‐old man presented to our clinic with severe dyspnea. At initial presentation, massive carcinomatous pleuritis and pericarditis were observed, and a lobulated mass in the anterior mediastinum was found on computed tomography. Cytological examination revealed adenocarcinoma accompanied by signet ring cells; however, his tumor showed aggressive growth without any possibility of treatment, and he died as a result of cancer progression within one month of admission. An autopsy confirmed thymic adenocarcinoma showing various histological features including mucinous, signet ring cell‐like, and trabecular features. Immunohistochemically, the tumor cells were positive for cytokeratin (CK) (AE1/AE3) but negative for TTF‐1. In addition, some tumor cells were positive for CD5 and KIT. Further examination revealed that tumor cells of the nonmucinous type were positive for CK7, and negative for CK20 and caudal‐type homeobox 2 (CDX2). The tumor cells with mucinous and signet ring‐like features were positive for CK20 and CDX2 and negative for CK7, indicating enteric differentiation. In particular, tumor cells with signet ring cell‐like features indicated widespread lymphangitic carcinomatosis and pulmonary tumor thrombotic microangiopathy (PTTM). The presence of signet ring cell‐like features with enteric differentiation is suggestive of a fulminant clinical course due to widespread lymphangiosis carcinomatosa and PTTM in patients with thymic adenocarcinoma.

**Key points:**

- Thymic adenocarcinoma is an extremely rare neoplasm.
- Histological features of thymic adenocarcinoma include mucinous, signet ring cell‐like, and trabecular features.
- Tumor cells with signet ring cell‐like features indicate widespread lymphangitic carcinomatosis and pulmonary tumor thrombotic microangiopathy.
- The presence of signet ring cell‐like features with enteric differentiation is associated with a fulminant clinical course.

## Graphical abstract

Thymic adenocarcinoma is an extremely rare neoplasm with various histological features including mucinous, signet ring cell‐like, and trabecular features. Tumour cells with signet ring cell‐like features indicate widespread lymphangitic carcinomatosis and pulmonary tumour thrombotic microangiopathy. The presence of signet ring cell‐like features with enteric differentiation is associated with a fulminant clinical course.

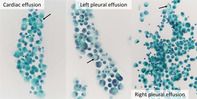

## Introduction

Thymic carcinoma is a rare neoplasm, and histologically, it is predominantly identified as squamous cell carcinoma. Because of the rarity of this entity, there is no established treatment for patients with unresectable thymic cancer. However, adenocarcinoma of the thymus is extremely rare, and its clinical features and pathogenesis are still unclear. To date, approximately 60 cases of primary thymic adenocarcinoma have been described in the English literature,^1^, ^2^, ^3^ and approximately 4.0% of thymic carcinomas have been identified as thymic adenocarcinomas.^4^ In previous studies, the presence of focal signet ring cell‐like features has been reported in only four cases.^1^, ^3^, ^4^ However, little is known about the clinicopathological behavior of thymic signet ring cells because of the lack of tumor specimens. Three of these four cases were defined as adenocarcinoma, not otherwise specified (NOS) and mucinous carcinoma, and only one case was defined as thymic adenocarcinoma with signet ring cell‐like features.^1^, ^3^, ^4^ In the case of the latter, the patient died immediately because of disease progression without any treatment a few days after admission.^3^ Herein, we present an autopsy case of thymic adenocarcinoma with signet ring cell‐like features and aggressive clinical course.

### Case report

**Figure 1:**
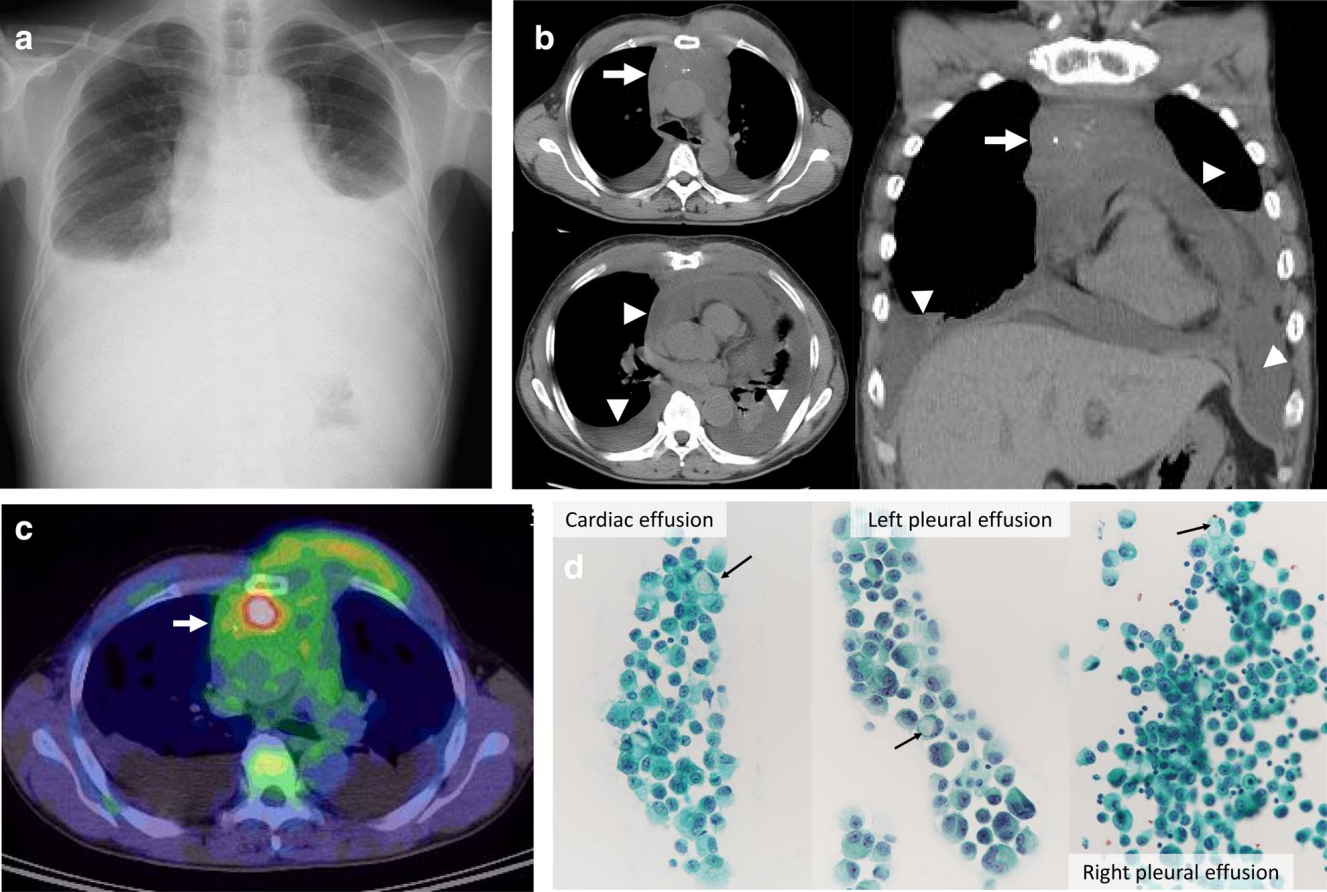
(**a**) Chest radiograph showing bilateral pleural effusions and cardiac enlargement. (**b**) Chest computed tomography (CT) scan revealed a lobulated mass measuring 87 × 83 mm in the anterior mediastinum (white arrow) with bilateral pleural effusion and cardiac effusion (white arrowhead). (**c**) Positron emission tomography (PET) imaging showed increased accumulation of 2‐deoxy‐2‐[18F] fluoro‐D‐glucose (^18^F‐FDG) in the anterior mediastinal mass with a maximal standardized uptake value (SUV_max_) of 7.9 (white arrow). (**d**) Cytological examination of bilateral pleural effusions and pericardial effusion revealed adenocarcinoma with signet ring cell‐like features (black arrows).

**Figure 2:**
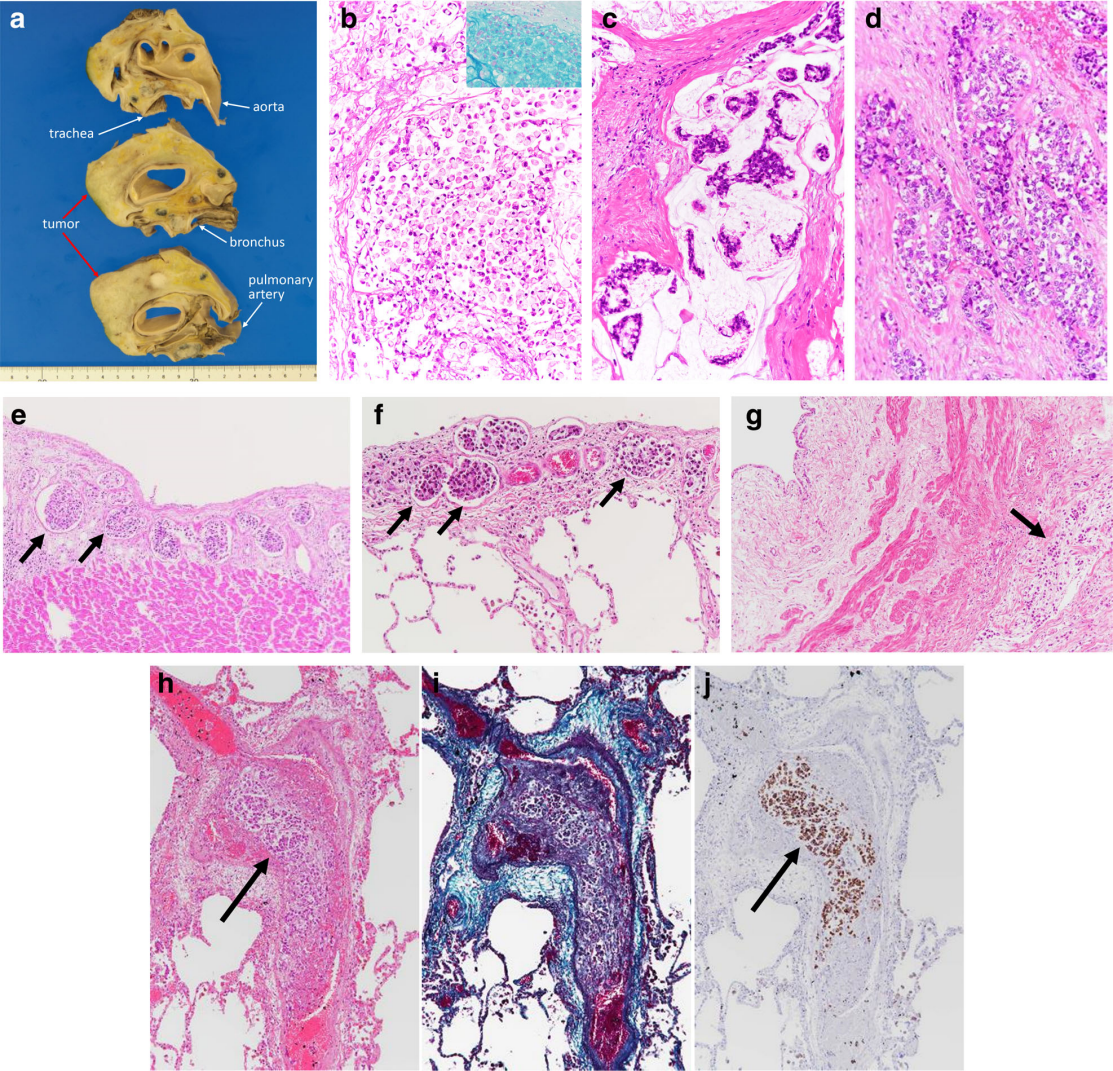
The axial cut surfaces of the tumor revealed an anterior mediastinal solid mass involving the aorta, pulmonary artery, trachea and bronchus. (**a**) No cystic lesions were identified on investigation. Histology of the anterior mediastinal mass revealed adenocarcinoma of the thymus with (**b**) heterogeneous mucinous pattern (inset figure; alcian blue pH2.5 staining); (**c**) signet ring cell‐like features; and (**d**) and glandular patterns. The tumor cells had spread to the (**e**) pericardium, (**f**) pleura; and (**g**) right ureteral lymphatics and were visible as clusters of predominantly signet ring cells (black arrows). Pulmonary tumor thrombotic microangiopathy (PTTM) was observed on (**h**) hematoxylin (black arrow) and eosin staining; (**i**) Elastica‐Masson staining; and (**j**) CK20 positive staining (black arrow).

A 52‐year‐old man presented at our clinic complaining of progressive dyspnea and lower‐limb edema for one month. Because of obvious orthopnea, he was immediately referred to the emergency department of our institution. On admission, his physical examination revealed excessive swelling of the bilateral jugular veins, cardiac murmur, pulmonary rales, and bilateral lower limb edema without any consciousness disorder. Laboratory examination showed a carcinoembryonic antigen of 51.2 ng/mL, carbohydrate antigen 19–9 of 115.7 U/mL, and D‐dimer of 12.5 μg/mL. Bilateral pleural effusions and cardiac enlargement were found on chest radiography (Fig 1a). A chest computed tomography (CT) scan revealed the presence of an anterior mediastinal mass measuring 10 × 9 × 6 cm in size, with bilateral pleural effusion and cardiac effusion (Fig 1b). Positron emission tomography (PET) imaging demonstrated increased accumulation of 2‐deoxy‐2‐[18F] fluoro‐D‐glucose in the anterior mediastinal mass (Fig 1c). A definitive diagnosis of adenocarcinoma with signet ring cell‐like features was made following thoracentesis and pericardiocentesis (Fig 1d). However, his general condition progressively worsened, and there was evidence of acute renal failure due to hydronephrosis and carcinomatous peritonitis, and he died within one month of admission. To elucidate the evaluation of cancerous spreading and primary site, a post mortem was performed assisted by an anatomical pathologist under the informed consent of the patient's family. The final diagnosis of the anterior mediastinal mass was proven to be adenocarcinoma of the thymus with heterogeneity of mucinous, signet ring cell‐like, and nonmucinous patterns, showing macroscopic findings (Fig 2a–d). The origin of this tumor was from the thymus. Mucinous component was predominant over nonmucinous component. Immunohistochemically, the tumor cells were positive for cytokeratin (CK) (AE1/AE3) but negative for TTF‐1. In addition, some tumor cells were positive for CD5 and KIT. Further examinations revealed that nonmucinous tumor cells were positive for CK7 and negative for CK20 and caudal type homeobox 2 (CDX2). On the other hand, the tumor cells with mucinous and signet ring‐like features were positive for CK20 and CDX2, and negative for CK7, indicating enteric differentiation. In particular, the tumor cells with signet ring cell‐like features had spread to the pericardium (Fig 2e), pleura (Fig 2f), and ureteral lymphatics (Fig 2g). Of note, the presence of pulmonary tumor thrombotic microangiopathy (PTTM) was confirmed (Fig 2h–j). Additionally, the tumor cells led to metastasis in the lymph nodes, retroperitoneum, pelvis, vertebra, and diaphragm. There was no evidence of a primary site in the gastrointestinal tract or other organs. With regard to the heterogeneous components of adenocarcinoma, the tumor cells with signet ring cell‐like features showed widespread lymphangitic carcinomatosis and PTTM. Hence, the patient died of multiple organ failure within one month from initial diagnosis.

## Discussion

Although only one report has previously discussed the pathological findings of thymic adenocarcinoma with signet ring cell‐like features,^3^ thoracentesis and mediastinal mass biopsy were performed to obtain tumor specimens, but not radical resection or autopsy. However, there was very little information regarding the other three cases. Thus, adequate pathological analysis of the primary site was limited and it was not possible to pathologically obtain evidence on the spread and infiltration of cells to the other organs apart from the thymus. To our knowledge, our report is an interesting case that elucidates tumor aggressiveness and histological behavior of thymic adenocarcinoma with signet ring cell‐like features based on post mortem pathological anatomy. Although our case included heterogeneous cells with mucinous, signet ring cell‐like, and nonmucinous features, tumor cells with signet ring cell‐like features are predominantly associated with infiltration into vessels and lymphatics. Moreover, PTTM was also observed. In the present case, the tumor cells with mucinous and signet ring cell‐like features were positive for CD20 and CDX2, respectively, indicating enteric differentiation. Thymic mucinous adenocarcinomas with enteric differentiation have been previously reported in 17 cases who underwent radical resection followed by radiation or chemotherapy.^1^, ^2^, ^3^, ^4^, ^5^, ^6^ Conversely, thymic adenocarcinoma with signet ring cell‐like features have been found to be negative for CK20 and CDX2 and positive for CK7,^3^ suggesting controversial results in our case. Our case was thought to include the component of signet ring cell‐like features with enteric differentiation. Moreover, tumor cells with signet ring cell‐like features were predominantly observed in all infiltrative and metastatic lesions. We believe that signet ring cell‐like features encourage tumor spread and invasiveness according to the findings of our post mortem examination.

PTTM is generally found at autopsy, and it is a fatal disease process related to pulmonary hypertension.^7^ A recent review discussed the clinical characteristics of 160 patients with PTTM, and the most frequent malignancy was gastric adenocarcinoma (59%), breast cancer (10%), lung cancer (6.3%), urothelial carcinoma (3.8%), and ovarian carcinoma (2.5%).^7^ In this review, hypoxemia, elevated D‐dimer level, and abnormal CT findings of ground‐glass opacities (GGO) were noted in 95%, 95%, and 82% of the cases, respectively. However, PTTM is difficult to diagnose, and most patients are diagnosed post mortem. However, little is known about the presence of PTTM due to thymic carcinoma. Thus, our case is the first to show manifestation of PTTM due to thymic adenocarcinoma. At the first manifestation in our patient, severe dyspnea and elevation of D‐dimer level were observed but not GGO findings on CT scan; however, the patient may have already experienced PTTM. Although the detailed mechanism of PTTM in patients with thymic cancer remains unclear, the histological component of a signet ring cell‐like feature with colonic differentiation seemed to be closely associated with its occurrence.

In conclusion, the presence of signet ring cell‐like features with enteric differentiation may encourage tumor spread, invasiveness, and PTTM in patients with thymic adenocarcinoma. Physicians should be alert to the possibility of clinical aggressiveness in thymic adenocarcinoma.

## Disclosure

The authors declare no conflict of interests.
